# Alveolar Abscess

**Published:** 1887-05

**Authors:** 


ARTICLE III.

### ALVEOLAR ABSCESS.

Whatever the cause, alveolar abscess is a suppurative inflammation of the periosteum, frequently resulting in the formation of a sac filled with pus at the apex of the root. In the early stages, the symptoms are simply those of periodontitis deep seated, but no great pain or tenderness on pressure, with frequently elongation, owing to the conical shape of the fang, the pressure resulting from the inflammation forcing the tooth out of its socket. If a patient were to come to you with the symptoms I have indicated, what would be your treatment? Manifestly, it would be your duty to do what you could to relieve the inflammation; but to do this intelligently, you must be sure of your diagnosis. First ascertain which is the suspected tooth. This is not usually difficult, as the tenderness will be a prominent system; then drill into the pulp cavity. Do this in that part of the crown which will render the subsequent treatment easy, by having direct access to the canal. If, when drilling, tenderness should manifest itself —for alveolar abscess is not always associated with a dead pulp—it may be desirable to suspend operations and have recourse to medicaments applied to the gum, near the end of the fang. I have not found any external treatment as valuable as the application of the dental tincture of iodine. This is made by adding an excess of the crystals of iodine to the B. P. tincture. After several months the alcohol takes up a much larger proportion of the iodine. Associated with this, or in lieu of it, a stimulant may be applied; and lately we have had furnished to us from the depots a small felt plaster containing capsicum. This is applied one or more times for several hours each, and will generally be found to give a good deal of relief. But generally sensitiveness is not met with, and the drill enters the pulp cavity. Frequently at this point pus or blood will make its escape, and the patient will express a feeling of relief; but this is not always the case. The pulp cavity is at times found to be quite empty; it is then your duty to try and reach the seat of the trouble by means of a long fine probe through the tooth. Sometimes the sac can readily be punctured in this way; but generally you must be satisfied with reaching the apical foramen. If no discharge takes place on removing the probe, it will then be desirable to drill out the canal through the apical foramen into the sac beyond.

Where the pulp is dead, whether you wish to treat a case of alveolar abscess or not, take the first opportunity to cone out the canals, open fully into the bulbous portion, and from that open into the canals, first using as large a bur as the cavity will allow, and then follow this with a small size; then use the large nerve canal drills and ream out, say half the length of the canal. You can readily see the object of this cone-shaped orifice; it is to facilitate the introduction of the medicine and the subsequent stopping of the root. When the orifice is fairly accessible, the best way to remove the *debris* from the canal is with a Swiss five-sided broach, the two smallest sizes made; this should have the point broken off, for a square point will, and a sharp point will not, allow you to push up the bit of cotton wood, or, what I consider far preferable, Japanese paper; the temper of the broach should be drawn by passing it quickly through the flame of a lamp, a blue color being what is most desired—in other words, a spring temper. The soft and extra soft nerve broaches of the depots I can find no use for. Wrap a minute bit of Japanese paper near the end of the broach and pass it into the canal, unwind and push it well in, then twist it on the broach again, withdraw and repeat. Sometimes it will take a dozen bits before all trace of discoloration disappears. This is equally necessary either for alveolar abscess or where it is desirable to prepare the canals for stopping.

For alveolar abscess, first fill the syringe with warm water, and after placing the point far up in the canal, wash it out by alternately compressing and releasing the rubber bulb; then draw all the water out and repeat with the peroxide of hydrogen. If bubbles appear, of course pus is present. Then introduce a solution of pure carbolic acid by means of the same syringe, filling the cavity near full; take a piece of softened red gutta percha, in bulk a little larger than the cavity, and by the use of a large flat-pointed instrument, nearly as large in diameter as the orifice, with considerable pressure force the carbolic acid, and if a fistula is present the carbolic acid will probably appear at the orifice. The presence of a fistula adds very materially to the probabilities of success, as it allows a free passage for the medicine through the seat of disease.

Should there be no fistula, the treatment is very much the same, except that, as it is difficult to force the carbolic acid into all parts of a closed cavity by the process mentioned, on account of the presence of air, resource must then be had to the fine probes, working the carbolic acid into the most remote parts. The cavity is then filled with

Japanese paper and the orifice stopped with paper soaked in sandarch. This ends the first dressing. The patient is directed, in case of pain, to remove the plug from the orifice. The second dressing is the same as the first; if a fistula is present, and you have been successful in forcing the medicine through it, it will in many cases be found to be closed, but two or more applications are generally necessary. Should it be impossible to force the carbolic acid through, resource must be had to either drilling through the nerve canal into the sac beyond, or to the production of an artificial fistula beyond by piercing the alveolus through the gum. This can be done with little pain by touching the gum with a crystal of carbolic acid before drilling, or preferably by the hypodermic use of cocaine, in which case efforts should be made to keep the fistula open by means of a piece of cotton wool until the treatment of the case is completed. It is seldom necessary to treat the tooth in this way many times; if the abscess does not readily yield to treatment, it will sometimes be found that persistent effort may cure it; but in these obstinate cases the tooth is often eventually lost. It will generally be found, however, by following the foregoing treatment, that eight out of ten cases will be successful.—ELLIOT, *Dental Record.*

